# Gene-environment interactions between *CREB1* and childhood maltreatment on aggression among male Chinese adolescents

**DOI:** 10.1038/s41598-022-05137-7

**Published:** 2022-01-25

**Authors:** Yanmei Zhang, Chun Kang, Haijun Yang, Min Yang, Sha Wei, Yan Wang, Xing Huang, Yizhen Yu

**Affiliations:** 1 Department of Preventive Medicine, School of Basic Medicine, Hubei University of Chinese Medicine, No. 16 Huangjiahu West Road, Hongshan District, Wuhan, 430065 China; 2 Department of Maternal, Child and Adolescent Health, School of Public Health, Tongji Medical College, Huazhong University of Science and Technology, Hangkong Road 13, Wuhan, 430030 Hubei China

**Keywords:** Neuroscience, Psychology, Health care, Medical research, Risk factors

## Abstract

Both the genetic and environmental factors may affect aggression susceptibility. However, the conclusions of these associations remain discrepant. In addition, studies that explored the association between *CREB1* and aggression were meager. The aim of our present study was to assess whether *CREB1* polymorphisms were related to aggression and also to explore the interactive effects of *CREB1* variants and childhood maltreatment on aggression. A total of 488 individuals with aggressive behavior and 488 controls were recruited. Aggression and childhood maltreatment were surveyed by standardized self-administered questionnaires. Buccal cells were also obtained and genotyping was conducted using SNPscan. Logistic regressions were applied to investigate both individual effects of *CREB1* polymorphisms and the interactive influences with childhood maltreatment on aggression. We found that adolescents who carried the rs4675690 T allele in *CREB1* showed a higher level of aggression compared with those who carried wildtype genotypes (CC) under the dominant model (*OR* = 1.67, 95% *CI*, 1.16–2.40) after controlling for age and childhood maltreatment. Moreover, we also found that rs4675690 T allele had a synergic additive interaction with childhood sexual abuse and emotional neglect on aggression. The significant interactive effects of *CREB1* polymorphisms and childhood maltreatment on aggression were reported for the first time.

## Introduction

Adolescent aggressive and violent behavior is a major public health issue with potentially negative influences on the physical and mental health of both victim and attacker^1,2^. Moreover, adolescent aggressive behavior has been linked to adverse late adolescent and adult consequences, such as intimate partner violence^3^, poor self- control^4^, and increased cyber-bullying^5^. Thus, aggression results in enormous economic and social burden, highlighting the urgent need for a better understanding of the specific biological mechanisms of aggression to help develop effective preventive measures^6^.

Tuvblad et al. had conducted a twin and adoption study, suggesting that approximately 50% of the variance of human aggression can be ascribed to the genetic factors, while the remaining 50% can be explained by the environmental factors^7^. A considerable body of studies have been conducted to evaluate the possible genetic background of human aggression. The effects of monoamine oxidase A gene (MAOA)^8^, catechol-O-methyltransferase gene (COMT)^9^, 5-hydroxytryptamine transporter gene (5-HTT)^10^ and dopamine receptor D4 gene (DRD4)^11^ on human aggression were studied most extensively and the vast majority of researches were conducted among mental patients. However, there was meager evidence with respect to the associations between the cyclic adenosine monophosphate responsive element binding protein 1 (*CREB1*) polymorphisms and aggression susceptibility. Furthermore, previous studies reported that boys tend to be more aggressive than girls^12^. Considering the availability of the study subjects’ buccal cells and the relatively limited funds, we only focused on boys in the current study.

In animal studies, the association between phosphorylation of the CREB, *CREB1* polymorphisms and aggression or major depressive disorder (MDD) had been reported^13^. In humans, the *CREB1* gene is located on chromosome 2, and it encodes the cyclic adenosine phosphate reactive element binding protein (CREB), which is broadly expressed in the human brain and becomes active only when phosphorylated^14^. Given CREB plays a crucial role in neuronal signal pathway, emotional reactivity, as well as reward and aversion circuits^15–18^, it is reasonable to explore the associations between *CREB1* polymorphisms and aggression susceptibility. Moreover, a line of researches claimed that *CREB1* had also been implicated in mood disorders^19^, antidepressant response, and suicide^13^, which all contribute to aggression^20^. However, to date, few studies examine the association between *CREB1* polymorphisms and human aggression.

With respect to the environmental factors on aggressive behaviors, a number of researchers had explored the complex associations between family adversity^21^, family income^22,23^, neighborhood disadvantage^24^, violent media exposure^25^, alcohol consumption^26,27^, and childhood maltreatment^28,29^ and aggression risk. However, the results and conclusions remained discrepant^22,23^. Additionally, to date, a low number of studies has investigated the interactive effects of *CREB1* polymorphisms and adverse environmental factors on aggression susceptibility.

The association between *CREB1* and childhood maltreatment was not found. However, Hasler’s^30^ research revealed that *CREB1* modulated the influence of childhood sexual abuse on adult's anger traits. Additionally, Li et al.^31^ conducted a systematic review and found that the interplay of childhood maltreatment with CREB1 variations significantly increased the risk of depression. In our current study, only the information of childhood maltreatment of environment-related factors was obtained by CTQ questionnaire. And the association between childhood maltreatment and aggressive behavior was statistically significant^32^.

Therefore, our aim was to examine the possible associations between polymorphisms in *CREB1* gene and the risk of aggression. Moreover, we also want to determine whether these significant polymorphisms showed interactive influences with childhood maltreatment on aggression in male Chinese adolescents.

## Results

### participants’ baseline characteristics

Table 1 shows the participants’ primary information in terms of aggression cases and controls. A total of 976 participants (488 cases and 488 controls) were recruited in the present study. The BWAQ total *T* scores in aggression cases and controls were 64.00 ± 7.57 points and 36.92 ± 9.71 points respectively. Compared to control individuals, subjects in the case group showed higher total scores of CTQ scale as well as its five subscales’ scores consisting of childhood physical abuse, sexual abuse, emotional abuse, physical neglect, and emotional neglect (All *P* < 0.05). Subjects living in the family with very low monthly income per person [< 1000 yuan (157 dollars)] and in the non-single-child families were more likely to be aggressive (All *P* < 0.05).

**Table 1:** Baseline characteristics of participants in the current case–control study.

Characteristics	Cases (n = 488)	Controls (n = 488)	*χ*^*2*^*/t*	*P*
Age (y) (mean ± SD)	18.35 ± 3.47	16.86 ± 3.54	8.54	< 0.05
**Single-child family**			9.05	< 0.05
No (%)	317(65.0)	271(55.5)		
Yes (%)	171(35.0)	217(44.5)		
**Family monthly income per person**			18.44	< 0.05
< 157 $ (%)	89(18.2)	46(9.4)		
157–314 $ (%)	369(75.6)	420(86.1)		
315–628 $ (%)	22(4.5)	15(3.1)		
> 628 $ (%)	8(1.6)	7(1.4)		
CTQ sum score	49.10 ± 16.15	35.10 ± 8.65	16.57	< 0.05
Emotional abuse	9.34 ± 4.77	6.03 ± 2.50	13.59	< 0.05
Emotional neglect	14.01 ± 5.71	10.57 ± 5.21	9.67	< 0.05
Physical abuse	8.88 ± 4.84	6.00 ± 3.00	11.61	< 0.05
physical neglect	9.74 ± 4.01	7.48 ± 2.64	10.36	< 0.05
Sexual abuse	7.63 ± 4.12	5.5 ± 2.55	9.32	< 0.05
BWAQ crude total score	98.16 ± 12.77	52.22 ± 5.54	72.88	< 0.05
PHY	13.66 ± 6.79	9.98 ± 1.83	11.54	< 0.05
VER	11.37 ± 3.47	9.31 ± 2.22	11.04	< 0.05
ANG	13.44 ± 6.17	11.48 ± 2.28	6.57	< 0.05
HOS	14.39 ± 7.39	12.52 ± 2.70	5.23	< 0.05
IND	11.91 ± 4.30	8.92 ± 2.06	13.86	< 0.05
BWAQ total *T* score	64.00 ± 7.57	36.92 ± 9.71	48.58	< 0.05

Variables are presented as mean ± SD for numerical data and number (%) for categorical data.

*CTQ* Childhood Trauma Questionnaire, *BWAQ* Buss and Warren’s Aggression Questionnaire, *y* years, *PHY* physical aggression, *VER* verbal aggression, *ANG* anger, *HOS* hostility, *IND* indirect aggression.

### Association analysis between individual SNP and aggression risk

Three single nucleotide polymorphisms (SNPs) namely rs4675690, rs7569963 and rs7594560 in *CEB1* of all subjects were successfully genotyped and specific information about these SNPs were exhibited in Table 2. The genotyping call rate of all SNPs were > 97% and the genotypes distribution in control group of our study conformed to Hardy–Weinberg equilibrium (HWE, All *P* > 0.05). The call rate is the percentage of successful genotype calls per passing SNP. And the minor allele frequencies (MAFs) of these three SNPs in our control subjects were similar to the corresponding data in the 1000 genome database of Han Chinese in Beijing, China (CHB). Furthermore, these three SNPs were not in strong LD with each other in the controls.

**Table 2:** Basic information of the SNPs in the current study.

Gene	rs ID	Position	Alleles	Minor allele	MAF in controls	MAF in CHB*	Call rate (100%)	*P* for HWE
*CREB1*	rs4675690	2:208,507,807	C/T	C	0.41	0.47	99.8	0.39
	rs7569963	2:208,473,184	G/A	G	0.11	0.13	97.8	0.55
	rs7594560	2:208,505,879	T/C	T	0.17	0.15	99.6	0.67

*MAF* minor allele frequency, *CHB* Han Chinese in Beijing, China, *HWE* Hardy–Weinberg equilibrium.

*MAF was downloaded from the online database of 1000 Genomes for Han Chinese in Beijing, China. These three SNPs are not in the coding region of CREB1.

As shown in Table 3, subjects who carried the rs4675690 CT genotype in *CREB1* had a higher risk of aggression compared with those who carried CC genotype (*OR* = 1.64, 95% *CI*, 1.12–2.39, *P* = 0.010). Subjects in case group were older than those in control group (*P* < 0.05). Furthermore, participants in case group obtained higher CTQ sum score than those in control group. Thus, we adjusted the effects for age and CTQ sum scores to increase comparability between the two groups. After adjusting for the effect of age and CTQ sum score, the difference remained significant (*OR* = 1.68, 95% *CI*, 1.14–2.47, *P* = 0.008). Similarly, compared with those who carried CC genotype, subjects who carried the rs4675690 TT genotype in *CREB1* had a higher level of aggression (*OR* = 1.57, 95% *CI*, 1.06–2.32, *P* = 0.024). After adjusting for age and CTQ sum score, the differences were still statistically significant (*OR* = 1.66, 95% *CI*, 1.11–2.47, *P* = 0.013). Subjects who carried the rs4675690 T allele in *CREB1* had a higher risk of aggression compared with those who carried CC genotype under the dominant model (*OR* = 1.61, 95% *CI*, 1.12–2.30,* P* = 0.009). After adjusting for age and CTQ sum score, their significant differences were also obtained (*OR* = 1.67, 95% *CI*, 1.16–2.40, *P* = 0.006). In the present study, three SNPs of *CREB1* gene were examined for their association with aggression risk, and the *P* < 0.05/3≈0.017 was considered to be statistically significant according to the multiple correction of Bonferroni. After the multiple correction, the presented *P* values under the dominant model (*P* = 0.009, and *P*^a^ = 0.006) remained to be statistically significant. Unfortunately, the other two markers, namely rs7569963 and rs7594560 were not found to be statistically significant.

**Table 3:** Association between SNPs in *CREB1* and aggression risk.

Gene	SNP	Genotype	Cases	Controls	*OR* (95% *CI*)	*P*	*OR* (95% *CI*)^a^	*P*^a^
*CREB1*	rs4675690	CC	58(11.9)	87(17.8)	1.00		1.00	
		CT	248(50.8)	227(46.5)	1.64(1.12–2.39)	0.010*	1.68(1.14–2.47)	0.008*
		TT	182(37.3)	174(35.7)	1.57(1.06–2.32)	0.024	1.66(1.11–2.47)	0.013*
		Dom			1.61(1.12–2.30)	0.009*	1.67(1.16–2.40)	0.006*
		Rec			1.07(0.83–1.39)	0.595	1.11(0.85–1.45)	0.433
		Add			1.18(0.98–1.42)	0.083	1.21(1.00–1.46)	0.047
*CREB1*	rs7569963	GG	36(7.4)	32(6.6)	1.00		1.00	
		GA	47(9.6)	43(8.8)	1.11(0.66–1.87)	0.69	1.20(0.65–2.21)	0.57
		AA	405(83.0)	413(84.6)	0.98(0.59–1.63)	0.94	1.04(0.57–1.90)	0.91
		Dom			1.03(0.63–1.70)	0.90	1.10(0.61–1.99)	0.75
		Rec			0.90(0.70–1.16)	0.40	0.89(0.66–1.19)	0.44
		Add			0.94(0.77–1.15)	0.54	0.94(0.74–1.20)	0.63
*CREB1*	rs7594560	TT	52(10.7)	50(10.2)	1.00		1.00	
		CT	70(14.3)	66(13.5)	0.99(0.72–1.39)	0.98	0.95(0.64–1.41)	0.81
		CC	366(75.0)	372(76.3)	1.04(0.73–1.48)	0.84	1.25(0.82–1.91)	0.30
		Dom			1.01(0.74–1.38)	0.94	1.06(0.73–1.53)	0.76
		Rec			1.04(0.79–1.36)	0.78	1.29(0.94–1.78)	0.12
		Add			1.02(0.86–1.22)	0.82	1.14(0.92–1.40)	0.23

*Dom* dominant model, *Rec* recessive model, *Add* additive model.

^a^Adjusted by age, total maltreatment score.

*Statistically significant.

### Gene-environment interactions with respect to aggression risk

Table 4 shows the results of our multiplicative and additive interaction influences between childhood maltreatment and rs4675690 polymorphism on the aggression risk. Compared with CC genotype carriers without experiences of sexual abuse, there were higher risks of aggression among T allele carriers without experiences of sexual abuse, CC genotype carriers with experiences of sexual abuse, and CT or TT genotype carriers with experiences of sexual abuse. The *OR*s were 1.56 (95% *CI*, 1.03–2.36), 3.53 (95% *CI*, 1.45–8.64) and 10.83 (95% *CI*, 6.01–19.49) respectively. In our current study, no statistically significant multiplicative interaction was found (*P* = 0.189). However, we found childhood sexual abuse had a synergic additive interaction with rs4675690 polymorphism in *CREB1* on aggression risk. The relative excess risk due to interaction (*RERI*), the attributable proportion due to interaction (*AP*) and the synergy index (*S*), were 6.74 (95% *CI*, 0.73–12.74), 0.62 (95% *CI*, 0.31–0.94) and 3.18 (95% *CI*, 1.10–9.24), respectively.

**Table 4:** Interactions between childhood maltreatment and rs4675690 on the risk of aggression.

Variables	rs4675690	Cases	Controls	*OR*(95%*CI*)^a^	*P*_*mul*_^a^	*RERI*(95%*CI*)	*AP*(95%*CI*)	*S*(95%*CI*)
**Physical abuse**					0.755	2.41 (-8.37–13.20)	0.21 (-0.67–1.09)	1.29 (0.38–4.44)
Negative	CC	40 (8.2)	83 (17.00	1.00				
Negative	CT/TT	288 (59.0)	378 (77.5)	1.65 (1.10–2.49)				
Positive	CC	18 (3.7)	4 (0.8)	8.66 (2.73–27.48)				
Positive	CT/TT	142 (29.1)	23 (4.7)	11.73 (6.54–21.05)				
**Sexual abuse**					0.189	6.74 (0.73–12.74)*	0.62 (0.31–0.94)*	3.18 (1.10–9.24)*
Negative	CC	40 (8.2)	78 (16.0)	1.00				
Negative	CT/TT	291 (59.6)	378 (77.5)	1.56 (1.03–2.36)				
Positive	CC	18 (3.7)	9 (1.8)	3.53 (1.45–8.64)				
Positive	CT/TT	139 (28.5)	23 (4.7)	10.83 (6.01–19.49)				
**Emotional abuse**					0.755	8.30 (-7.12–23.71)	0.49 (-0.19–1.18)	2.11 (0.47–9.46)
Negative	CC	45 (9.2)	84 (17.2)	1.00				
Negative	CT/TT	339 (69.5)	392 (80.3)	1.70 (1.14–2.52)				
Positive	CC	13 (2.7)	3 (0.6)	7.82 (2.11–29.02)				
Positive	CT/TT	91 (18.6)	9 (1.8)	16.81 (7.71–36.64)				
**Physical neglect**					0.508	1.68 (-0.23–3.58)	0.38 (-0.01–0.76)	1.95 (0.73–5.16)
Negative	CC	36 (7.5)	68 ( (14.0)	1.00				
Negative	CT/TT	224 (46.4)	311 (64.0)	1.46 (0.93–2.28)				
Positive	CC	21 (4.3)	18 (3.7)	2.31 (1.08–4.96)				
Positive	CT/TT	202 (41.8)	89 (16.3)	4.45 (2.74–7.21)				
**Emotional neglect**					0.156	2.61 (0.67–4.54)*	0.52 (0.24–0.81)*	2.90 (1.01–8.49)*
Negative	CC	34 (7.2)	63 (13.3)	1.00				
Negative	CT/TT	221 (47.1)	311 (65.6)	1.42 (0.90–2.25)				
Positive	CC	21 (4.5)	22 (4.6)	1.95 (0.93–4.10)				
Positive	CT/TT	193 (41.2)	78 (16.5)	4.98 (3.01–8.24)				
**Total CTQ**					0.133	2.40 (0.68–4.12)*	0.47 (0.19–0.76)*	2.44 (0.99–6.00)
Negative	CC	23 (4.7)	55 (11.3)	1.00				
Negative	CT/TT	122 (25.0)	256 (52.5)	1.14 (0.67–1.94)				
Positive	CC	34 (7.0)	32 (6.6)	2.54 (1.28–5.04)				
Positive	CT/TT	309 (63.3)	145 (29.7)	5.10 (3.01–8.62)				

*P*_mul_ was calculated using the multiplicative interaction term.

^a^Adjusted by age.

*Statistically significant.

Furthermore, we also found childhood emotional neglect had a synergic additive interaction with rs4675690 polymorphism in *CREB1* on aggression risk. The *RERI*, *AP* and *S* were 2.61 (95% *CI*, 0.67–4.54), 0.52 (95% *CI*, 0.24–0.81) and 2.90 (95% *CI*, 1.01–8.49), respectively.

**Figure 1:**
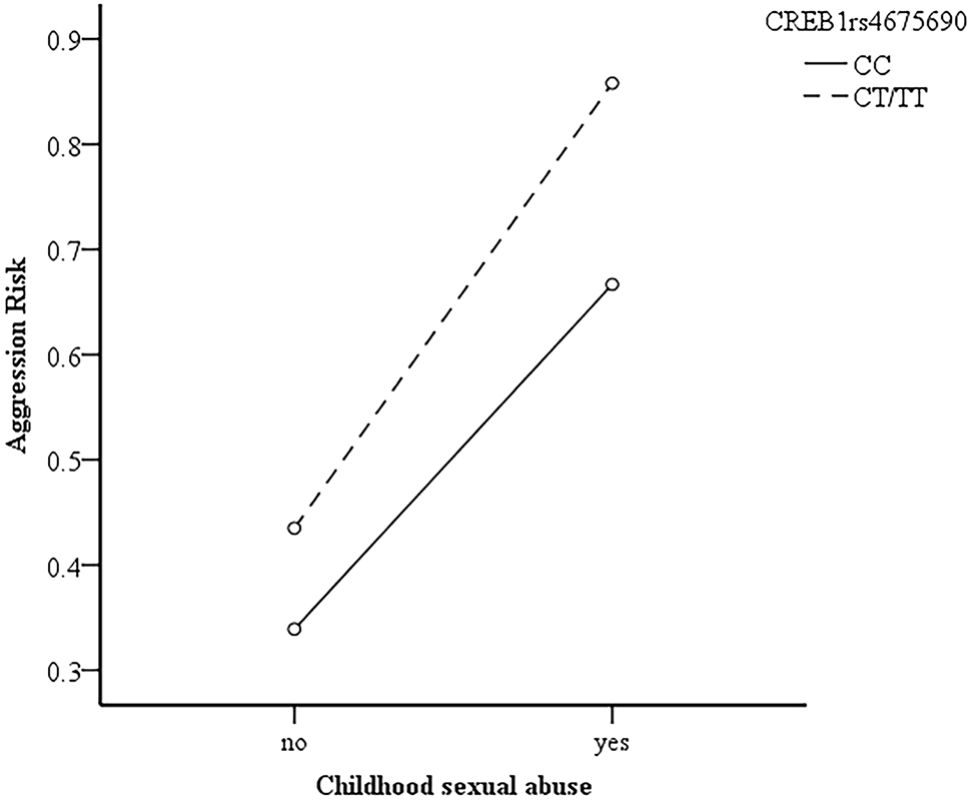
The interactive effect of CREB1 rs4675690 polymorphism and childhood sexual abuse on aggression risk.

**Figure 2:**
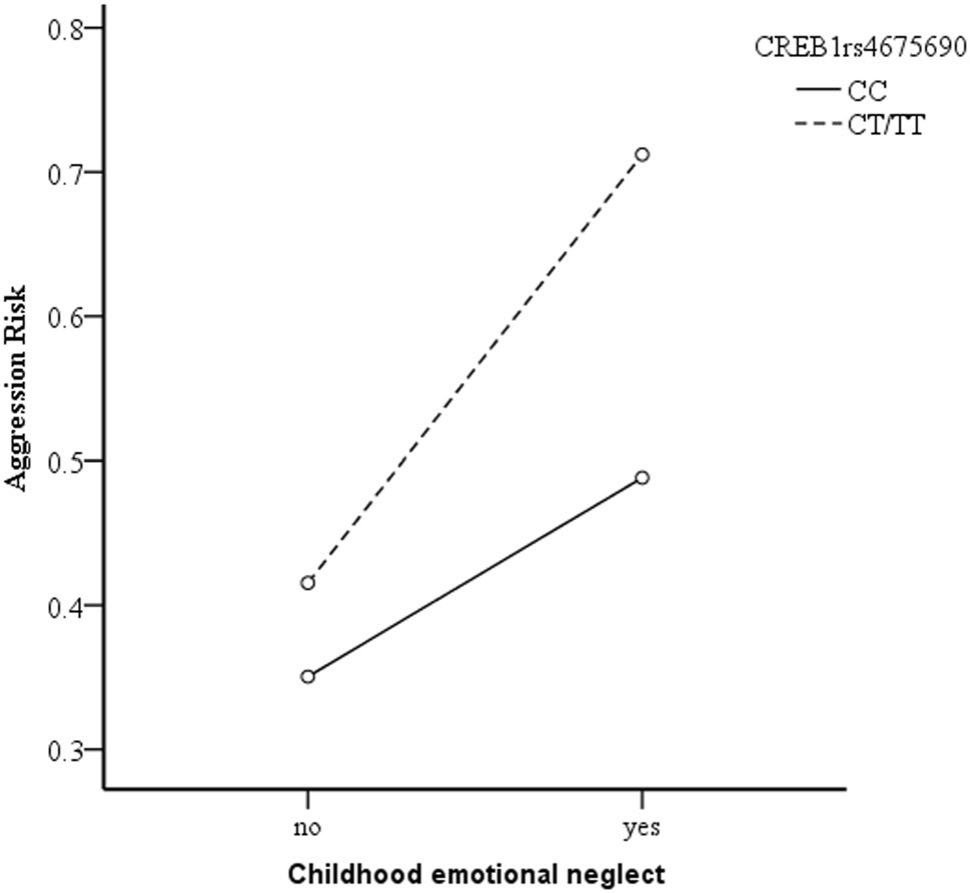
The interactive effect of *CREB1* rs4675690 polymorphism and childhood emotional neglect on aggression risk.

As shown in Fig. 1 and Fig. 2, whether or not the subjects had a history of childhood sexual abuse and childhood emotional neglect, subjects who carried the rs4675690 CT or TT genotype in *CREB1* showed a higher level of aggression compared with those who carried CC genotype. However, the effect of rs4675690 polymorphism in *CREB1* on aggressive risk had differences according to whether or not the subjects had a history of childhood sexual abuse and childhood emotional neglect.

## Discussion

In this case–control study, we investigated the associations between *CREB1* genetic variants and the risk of aggression. Our results suggested that *CREB1* rs4675690 T allele was significantly associated with an increased risk of aggression. Potential synergic additive interactions between the rs4675690 T allele and childhood sexual abuse and childhood emotional neglect were also found. To the best of our knowledge, this is the first study to demonstrate interactive effects between *CREB1* rs4675690 polymorphism and childhood sexual abuse and childhood emotional neglect on aggression susceptibility.

A previous study using functional magnetic resonance imaging (fMRI) suggested that the rs4675690 polymorphism is associated with the modulation of neural responses to negative stimuli^33^. Fortier et al.^33^ showed that children carrying the TT genotype, compared to those carrying the CC genotype might increase later in life susceptibility to emotional dysregulation and depressive symptoms by using fMRI. Furthermore, a recent study showed that emotional dysregulation may contribute to aggressive and violent behavior^34^. Therefore, our findings are consistent with the abovementioned results.

Several studies showed that acute or chronic stress activates the CREB signaling pathway, and stress events inhibit the function of CREB, reducing its total content in the body or reducing its phosphorylation activity^35^. Laifenfeld and his colleagues’ animal studies have also shown that chronic stress causes a decrease in phosphorylated CREB levels in the hippocampus, striatum, and frontal cortex regions^36^. Furthermore, Huang et al. conducted an animal study and found that the level of phosphorylated CREB in the hippocampus of rats lacking maternal love or deprived of maternal love was significantly reduced^37^. Additionally, in HaploReg database (http://archive.broadinstitute.org/mammals/haploreg/haploreg.php), we predict the site of rs4675690 in the column "QTLhits" shows "2 hits", and it indicated that the site may be the expression quantitative trait locis (eQTLs) of *CREB1* gene, and it may regulate the expression of *CREB1* gene. Westra et al.^38^ found that large-scale eQTL mapping could provide insight into the downstream effects of several trait-associated variants. Thus, the rs4675690 of “QTL hits” may affect the expression of *CREB1* gene. Nevertheless, the exact effect of rs4675690 polymorphism so far is uncertain, and it is also unknown whether it is functional or whether the predicted influence arises from another undiscovered functional marker. Until more concrete data is known about the functionality of the rs4675690 polymorphism, we would like to explain associations at the level of gene or its inner structures such as the promoter, exons, introns, and so on. Therefore, it is reasonable to assume that the polymorphism of *CREB1* rs4675690 affects the expression level of *CREB1* gene, thus affecting the level of CREB in vivo, and finally leading to the difference in the risk of aggression. Further studies with larger sample sizes and more rigorous design are certainly needed to confirm our findings.

With respect to the environmental factor, there is mounting evidence that early childhood maltreatment will cause biochemical, structural, and functional changes in our brain^39–41^. Glaser and his colleagues^41^ published a review showing decreased hippocampal volume were found in children who experienced early trauma or abuse. Furthermore, Teicher’s research group^39^ showed that childhood maltreatment or abuse was related to a reduced adult hippocampal volume, particularly on the left side of the brain by using MRI. This decreased hippocampal volume may influence development of the dorsal lateral prefrontal cortex (DLPFC), which resulted in increased aggression in adults when malfunctional^40^. This may somewhat interpret the association between childhood maltreatment and aggression risk.

The interactive effects between childhood sexual abuse, childhood emotional neglect and rs4675690 polymorphism in *CREB1* gene on aggression risk could be understood at several different levels. First, individuals confronted with “stress-loaded environments and exposure” (such as childhood sexual maltreatment and childhood emotional neglect) would reduce their aggressive behavior when effective interventions are available. Another perspective revealed that beneficial genetic factors (low risk genotypes) could provide a protective effect on aggression risk, especially when in a bad circumstance. One possible reasonable understanding for the finding of significant interaction is that both rs4675690 polymorphism and childhood sexual abuse and emotional neglect affect the transcription and expression of *CREB1* gene, and then influence the risk for aggression. However, the exact mechanism of interaction ought to be confirmed by a number of strictly designed functional experiments. However, the childhood physical abuse, childhood emotional abuse, and childhood physical neglect were not found the statistically significant interactions. Subjects who have experienced childhood sexual abuse may feel ashamed and powerless. Childhood Sexual abuse would be associated with lifetime aggression even after control of co-occurring abuse (physical abuse, neglect)^42^. Childhood sexual abuse may have the greatest impact on aggressive behavior. This may partly explain the statistically significant interaction between childhood sexual abuse and rs4675690 polymorphism in *CREB1* on aggression risk. Additionally, childhood emotional neglect refers to the failure of caretakers to provide basic psychological and emotional needs including love, belonging, nurturance, and support. It has not been reported yet that childhood emotional neglect still has an impact on aggressive behavior after controlling for other forms of maltreatment. Subsequent studies with a larger sample size are pressing needed to confirm that childhood emotional neglect and *CREB1* polymorphism interact with aggressive behavior. Furthermore, the specific biological mechanism of the interactions also needs to be further studied.

Genetic and environmental interactive effects on aggression have been reported in a number of previous studies. For example, Cloninger et al.^43^ conducted a classic study of adoption design and found that adopted children with criminal biological parents and also reared by a family with bad environment was currently exhibited higher prevalence of antisocial and aggressive behavior than those with criminal biological parents but not raised in a family with bad environment, and also than those raised amid adversity but with normal biological parents. However, the adopted children not reared in adversity and not with criminal biological parents had the lowest risk for antisocial and aggressive behavior. Moreover, a representative cohort study with 1,116 twin pairs showed that the effects of maltreatment on conduct problems risk were stronger among those with a high genetic susceptibility for such problems compared with children at a low genetic risk^44^. In our current study, the rs4675690 polymorphism in *CREB1* gene was an instance of the genetic factor while childhood sexual abuse and childhood emotional neglect were instances of the early adverse environmental factors. Therefore, our conclusions of significantly additive interactive effects on aggressive behavior might be regarded as further support to the abovementioned studies.

Our present study has a number of certain strengths. First, this study has a relatively large sample size, standard questionnaires with good reliability and validity, efficient genotyping methods, and precisely assess of possible confounders. Second, the vast majority of previously aggression-related genetic studies were conducted among psychiatric patients. However, in our present study, subjects diagnosed with mental illness were all excluded, thus, our findings may be more representative and could be generalized to male adolescents. Third, all of our interviewers were professionally trained and blind to research hypothesis before undertaking this study. Thus, to the best of our knowledge, ascertainment bias was avoided as far as possible in this study. Fourth, the significantly interactive effects of rs4675690 polymorphism in *CREB1* gene and childhood sexual abuse and emotional neglect on aggression were reported the first time, especially among male Chinese adolescent.

This study also has several limitations that must be noted. First, only three SNPs in *CREB1* gene were genotyped, and then evaluated their associations with aggression risk. Thus, completely sequencing of *CREB1* gene should be needed to estimate the associations between *CREB1* polymorphisms and aggression risk in future studies. However, these three SNPs genotyped and analyzed in our present study were selected through rigorous procedure and they were also the mostly documented as being associated with aggression related behavior in previous researches^30,45^. Hence, considering the limited research funding, it remained worth and reasonable to explore the associations between these three SNPs and aggression. Second, merely male adolescents were included in our current research, thus, considering the possible sex differences, our results and conclusions may not be directly generalized to females. Third, only one-stage case–control study was conducted to evaluate the associations between *CREB1* polymorphisms and aggression risk. Our findings should be confirmed among another independent populations, therefore, the application of the current conclusions should be cautious. Finally, the RNA and protein expression of *CREB1* should definitely analyzed in future studies to check the biological impact of the SNPs.

## Conclusions

In conclusion, our current study found that rs4675690 T allele was a risk factor for aggression risk. Moreover, we first reported that significant additive interactive effects exist between rs4675690 polymorphism and childhood sexual abuse and emotional neglect on aggression susceptibility. Our findings may contribute to explain and understand the etiological mechanism of aggressive behavior, which may help to develop effective prevention strategies for aggressive behavior. Further studies with more participants and more rigorous design are certainly needed to confirm our findings.

## Materials and methods

### Study subjects

Within the ongoing adolescents’ aggression study project, a sample of 976 male subjects were included in the current study. This study was conducted from September 2014 to April 2019 in selected schools from Xiaogan, Yingshan and Wuhan cities in Hubei Province, China^46^. According to the score of the Buss-Warren aggression questionnaire, 488 male adolescents with high scores were selected as aggression cases. In terms of the principle of voluntary provision of buccal cells for genotyping, 488 participants were selected as control from those with low scores. And specific demarcation criteria are described in detail below. Furthermore, previous studies reported that boys tend to be more aggressive than girls^12^. Considering the availability of the study subjects’ buccal cells and the relatively limited funds, we only focused on boys in the current study.

A structured questionnaire was applied to collect information on basic demographic characteristics, family financial situation, history of childhood maltreatment, and other related information by a group of trained interviewers, who explained the procedure and purpose of this study. The self-administered survey was anonymous, and was completed in classrooms during a 30 to 40 min period. All subjects were informed that there were no so-called correct or incorrect answers, that all questions should be answered honestly and accurately, and that their answers would be kept private and used for scientific research merely. Participants with a diagnosis of schizophrenia or autism based on clinical records were excluded from our present study^46^. Additionally, Participants with chronic medical illness who were required to go to the hospital for regular examination and take special medications regularly or have dietary restrictions were excluded from our present study.

### Ethics statement

Written informed consent was obtained from all participants and their parents or guardians. This study was approved by the Ethics Committee of the School of Public Health, Tongji Medical College, Huazhong University of Science and Technology and the School of Basic Medicines, Hubei University of Chinese Medicine. All the methods in this study were carried out in accordance with the approved guidelines.

### Assessment of aggression

The Chinese version of Buss and Warren’s Aggression Questionnaire (BWAQ) was administered to evaluate aggression^47,48^. This self-answered questionnaire consisted of 34 items, which were reported on a 5-point Likert scale ranging from 1 (not at all like me) to 5 (completely like me). It measured five dimensions related to aggression: physical aggression (PHY), verbal aggression (VER), anger (ANG), hostility (HOS), and indirect aggression (IND), with the higher scores of each dimension reflecting corresponding higher levels of aggression. According to the interpretation of BWAQ, The crude total scores are first converted to total *T* scores and the *T* score is a standard score with a mean of 50 and a standard deviation of 10. The related aggression total *T* scores were classified into seven grades (1 to 7): very low (≤ 29* T*), low (30* T*–39* T*), low average(40* T*–44* T*), average (45* T*–55* T*), high average (56* T*–59* T*), high (60* T*–69* T*), and very high (≥ 70* T*) respectively. Participants were recognized as aggression cases if their total *T* scores classified into the sixth or seventh grade (≥ 60* T*). The controls selected from the ongoing aggression study project participants with BWAQ scores that below 60* T*. The BWAQ has been proven to have good psychometric properties^49^ (internal reliability: PHY = 0.81, VER = 0.71, ANG = 0.64, HOS = 0.61, and IND = 0.62). The internal consistency as reflected by the overall Cronbach coefficient alpha of the 34 items in the present study was 0.94, and the internal consistency for each subscale was 0.87, 0.62, 0.71, 0.75, and 0.78, respectively.

### Assessment of childhood maltreatment

All participants completed the short version of the Childhood Trauma Questionnaire (CTQ)^50,51^, a 28-item retrospective self-report questionnaire. In the CTQ, the child maltreatment domains of physical, sexual, and emotional abuse, and physical and emotional neglect are each assessed by five items that participants rate on a 5-point, Likert-type scale ranging from “never true” to “very often true” according to the frequency with which each event occurred. Physical abuse was defined as bodily assaults on a child by an adult or older person that poses a risk for or results in injury; sexual abuse as sexual contact, or conduct between a child younger than 18 years of age and an adult or older person; emotional abuse as “verbal assaults on a child’s sense of worth or well-being, or any humiliating, demeaning, or threatening behavior directed toward a child by an adult or older person”; physical neglect as failure of caregivers to provide a child’s basic physical needs including food, shelter, clothing, safety, and health care; and emotional neglect as the failure of caretakers to provide basic psychological and emotional needs including love, belonging, nurturance, and support^52^. Scores across the five child maltreatment domains were summed to achieve a total CTQ score; the five subscale scores permitted assessments to be made for effects in each of the five abuse and neglect domains. Each subscale score ranges from 5 to 25 points, with a subscale total score of more than 5 was defined as having experienced this type of neglect or abuse in childhood. None of the five subscale types of neglect or abuse was considered to have been excluded from total childhood maltreatment. Internal consistency has previously ranged from 0.76 to 0.92^11,51^, and was 0.82 in our current study.

### Identification of candidate single nucleotide polymorphisms (SNPs) and genotyping by SNPscan sequencing

The procedure for screening candidate *CREB1* SNPs were as follows. First, all validated SNPs in a region that included CREB1 and flanking regions were all identified using the 1000 Genomes database (http://www.internationalgenome.org/). Second, these SNPs were filtered by MAF for Han Chinese in Beijing of China (CHB) > 5% from this database. Third, The Tagger program (http://www.broad.mit.edu/mpg/tagger/), which examines pairwise and multiallelic linkage disequilibrium to determine the minimum set of SNPs necessary to capture all common genetic variation in a region^53^. SNPs in strong LD with each other (r^2^ > 0.80) were considered redundant and only one was reserved. As a consequence, a total of three SNPs remained for further analyses (rs4675690, rs7569963, and rs7594560).

Buccal cells were collected from participants by scraping the left and right inner cheeks 10 times with a buccal collection brush (Shenzhen Huachenyang Technology Company, China). The detached head of the collection brush was then placed into a 1.5 ml microcentrifuge tube with 300 μl cell lysis solution. Genomic DNA was extracted from buccal brushes using the Gentra Puregene Buccal Cell Kit (QIAGEN, Duesseldorf, Germany) according to the manufacturer’s instructions. The DNA concentrations were determined using a Nano-Drop 1000 spectrophotometer (Thermo Fisher Scientific, Waltham, Massachusetts, USA). The mean DNA concentration was 30 ng/μl and the total amount of DNA is more than 500 ng for the SNPscan in our present study.

Genotyping was performed for rs4675690, rs7569963, and rs7594560 in a total of 976 subjects. These three SNPs were genotyped using a custom-by-design 48-Plex SNPscan™ Kit (Cat#:G0104; Genesky Biotechnologies Inc., Shanghai, China). This kit was developed according to patented SNP genotyping technology by Genesky Biotechnologies Inc., which was based on double ligation and multiplex fluorescence PCR^54^. With the aim to validate the genotyping accuracy using SNPscan™ Kit, 5% duplicate samples were analyzed by single nucleotide extension using the Multiplex SNaPshot Kit (Applied Biosystems Inc., Foster City, CA, USA), and the concordance rates were more than 99%.

### Statistical analysis

The Hardy–Weinberg equilibrium for genotypes distribution among controls was assessed by a goodness-of-fit χ^2^ test. The χ^2^ *and student t* tests were used to compare qualitative variables and continuous data between case and control groups respectively. Association analysis between each SNP and aggression risk was carried out with unconditional logistic regression using genotype, dominant, recessive, and additive models. Odds ratios (*OR*s) and corresponding 95% confidence intervals (95% *CI*s) were estimated for the effects of SNPs on aggression predisposition. A likelihood ratio test in the logistic regression model was used to evaluate multiplicative and additive interactions. Synergy index (*S*), attributable proportion caused by interaction (*AP*), and the relative excess risk caused by interaction (*RERI*) and their 95% *CI*s were calculated in additive interaction analyses. In this study, we took the “both childhood maltreatment free (CM-free) and genotype of *CREB1* with low risk of aggression”as the reference group, *OR*_*11*_ refers to the effect of genotype with high risk of aggression in *CREB1* and with childhood maltreatment affected (CM-affected); *OR*_*10*_ represents the effect of genotype with high risk of aggression in *CREB1* but with CM-free; while *OR*_*01*_ is the effect for genotype with low risk of aggression in *CREB1* but with CM-affected. Then, *RERI* = *OR*_11_-*OR*_10_- *OR*_01_ + 1; *AP* = *RERI*/*OR*_*11*_; *S* = (*OR*_*11*_–1)/[(*OR*_*01*_–1) + (*OR*_*10*_–1)]. When a 95% *CI* of S containing 1 and a 95% *CI* of AP and *RERI* containing 0, then a lack of additive interaction was recognized. Main statistical analyses were performed on SPSS software (version 17.0). Linkage disequilibrium (LD) analysis was performed using Haploview v4.2 software. Additive interaction analysis was performed using the Excel sheet made by Andersson et al.^55^. All reported *P* values were two-sided, and *P* < 0.05 was considered to be statistically significant.

## Supplementary Information

Supplementary Information.

## Data Availability

The main data used in the present study were available in the supplementary information.

## Abbreviations

CREB: Cyclic adenosine monophosphate response element binding protein
PHY: Physical aggression
VER: Verbal aggression
ANG: Anger
HOS: Hostility
IND: Indirect aggression
CTQ: Childhood trauma questionnaire
SNP: Single nucleotide polymorphisms
